# Evolution of Dislocation Loops Induced by Different Hydrogen Irradiation Conditions in Reduced-Activation Martensitic Steel

**DOI:** 10.3390/ma11112276

**Published:** 2018-11-14

**Authors:** Weiping Zhang, Liping Guo, Zhenyu Shen, Jingping Xin, Qunying Huang, Yaxia Wei, Yunxiang Long, Xiong Zhou, Cheng Chen

**Affiliations:** 1 Hubei Key Laboratory of Nuclear Solid Physics, Key Laboratory of Artificial Micro- and Nano-structures of Ministry of Education and School of Physics and Technology, Wuhan University, Wuhan 430072, China; zhangweiping@whu.edu.cn (W.Z.); shenzy@whu.edu.cn (Z.S.); weiyx@whu.edu.cn (Y.W.); kobe-long@whu.edu.cn (Y.L.); peter.zhoux@whu.edu.cn (X.Z.); 2016202020013@whu.edu.cn (C.C.); 2 Key Laboratory of Neutronics and Radiation Safety, Institute of Nuclear Energy Safety Technology, Chinese Academy of Sciences, Hefei 230031, Anhui, China; jingping.xin@fds.org.cn (J.X.); qunying.huang@fds.org.cn (Q.H.)

**Keywords:** dislocation loops, hydrogen ion irradiation, transmission electron microscope (TEM), reduced-activation ferritic/martensitic steels

## Abstract

Hydrogen can be induced in various ways into reduced-activation ferritic/martensitic (RAFM) steels when they are used as structural materials for advanced nuclear systems. However, because of the fast diffusion of hydrogen in metals, the effect of hydrogen on the evolution of irradiation-induced defects was almost neglected. In the present work, the effect of hydrogen on the evolution of dislocation loops was investigated using a transmission electron microscope. Specimens of reduced-activation ferritic/martensitic (RAFM) steels were irradiated with hydrogen ions to 5 × 10^20^ H^+^ • m^−2^ at 523–823 K, and to 1 × 10^20^ H^+^ • m^−2^ − 5 × 10^20^ H^+^ • m^−2^ at 723 K. The experimental results reveal that there is an optimum temperature for dislocation loop growth, which is ~723 K, and it is greater than the reported values for neutron irradiations. Surprisingly, the sizes of the loops produced by hydrogen ions, namely, 93 nm and 286 nm for the mean and maximum value, respectively, at the peak dose of 0.16 dpa under 723 K, are much larger than that produced by neutrons and heavy ions at the same damage level and temperature. The results indicate that hydrogen could enhance the growth of loops. Moreover, 47.3% $\frac{1}{2}\;$a_0_ <111> and 52.7% a_0_ <100> loops were observed at 523 K, but $\frac{1}{2}\;$a_0_ <111> loops disappeared and only a_0_ <100> loops existed above 623 K. Compared with the neutron and ion irradiations, the presence of hydrogen promoted the formation of a_0_ <100> loops.

## 1. Introduction

A challenge for developing advanced nuclear systems, such as fusion reactors, Generation IV fission reactors, and accelerator-driven spallation (ADS) devices, is the deep understanding of the complicated irradiation damage mechanisms in irradiation resistant materials [1,2,3,4]. Reduced-activation ferritic/martensitic (RAFM) steels are considered as prime candidate structural materials for advanced nuclear systems, owing to their excellent mechanical properties, microstructural stability, and irradiation resistance [5,6,7]. However, the properties of the RAFM steels will degrade when they are exposed to conditions of high energy neutron irradiations, such as 14 MeV for fusion reactors and hundreds of MeV for ADS devices. In such conditions, hydrogen and helium are introduced via nuclear transmutation reactions of (n, p) and (n, α), which cause the degradations to be accelerated [8,9,10,11]. The production rate of hydrogen is 30–40 appm/dpa in fusion reactors and ~1500 appm/dpa in ADS devices. Hydrogen can also be introduced by fuel permeation and diffusion with a high concentration in fission and fusion reactors. In previous studies, there has been considerable experience in experimentation and modeling regarding the role that helium plays in the accelerated degradations of materials [12,13,14], but there has been little for hydrogen. Because the diffusion of hydrogen is fast, it was generally understood that hydrogen would diffuse out of steels, and that it had little effect on microstructure evolution, especially at elevated temperatures [4].

Actually, hydrogen played an important role in the microstructure evolution. Kupriiyanova et al. found that the co-implantation of hydrogen resulted in more swelling than heavy-ion implantation alone [7]. In RAFM steels, there are some studies concerning the role of hydrogen in microstructures, most of which are focused on bubbles [9,15], whilst there are few studies on dislocation loops. However, the dislocation loop is a type of nanoscale crystal defect that is the most easily produced by irradiation. Some experimental results have suggested that irradiation hardening mainly originates from the presence of dislocation loops [16]. Therefore, it is significant to investigate the evolution of loops induced by hydrogen irradiation. In the present study, we investigate the evolution of loops induced by hydrogen ion irradiation at different conditions (fluence from 1 × 10^20^ to 5 × 10^20^ H^+^ • m^−2^, and temperature from 523 to 823 K) in RAFM steels via transmission electron microscope (TEM) observation.

## 2. Materials and Methods

The composition of the steel used in present study was Fe 9.24 wt %, Cr 2.29 wt %, W 0.49 wt %, Mn 0.25 wt %, V 0.25 wt %, Si 0.088 wt %, and C 0.0059 wt % P. The details of the heat treatment and fabrication are reported in the literature [17]. The material was cut into sheets with a thickness of 0.5 mm. The sheets were mechanically polished to the thickness of 0.1 mm, from both sides. Disks with diameter of 3 mm were punched out from the sheets, and were then finally milled to a thickness of about 50 µm. The perforation thin foils for the TEM observation were done by twin-jet electro-polishing, using a solution of 5% perchloric acid and 95% ethanol electrolyte at 243 K.

**Figure 1:**
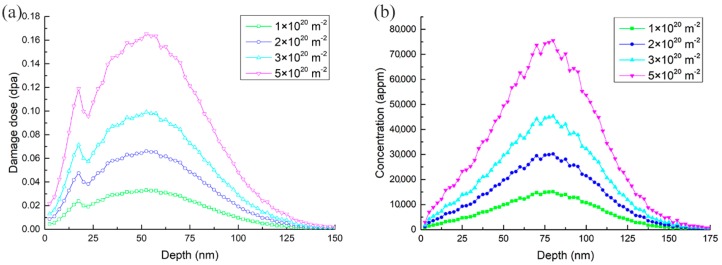
SRIM calculations of depth profiles of (**a**) damage events and (**b**) hydrogen concentrations in the specimen, irradiated using 10 keV H^+^ to 1 × 10^20^ H^+^ • m^−2^, 2 × 10^20^ H^+^ • m^−2^, 3 × 10^20^ H^+^ • m^−2^, and 5 × 10^20^ H^+^ • m^−2^.

One part of the specimens (prepared TEM foils) was irradiated by 10 keV H^+^ to 5 × 10^20^ H^+^ • m^−2^ at 523 K, 623 K, 723 K, and 823 K, respectively. At the temperature of 723 K, the other part of the specimens was irradiated by 10 keV H^+^ to 1 × 10^20^ H^+^ • m^−2^, 2 × 10^20^ H^+^ • m^−2^, 3 × 10^20^ H^+^ • m^−2^, and 5 × 10^20^ H^+^ • m^−2^, corresponding to the peak damage dose of 0.03 dpa, 0.06 dpa, 0.10 dpa, and 0.16 dpa, respectively. Figure 1 shows the atom concentration and damage dose profiles calculated using SRIM2008, with a displacement energy of 40 eV [18]. The irradiations were conducted using the ion implanter from the Accelerator Lab of Wuhan University. The observations of the microstructures were conducted using a JEM-2010HT TEM (JEOL, the Center for Electron Microscopy in Wuhan University, China) operated at 200 kV. The thickness (~120 nm) of the specimens at the observed area were estimated by counting the number of thickness fringes from the edge of the thin foil.

## 3. Results

### 3.1. Dislocation Loop Evolution in RAFM Steel with Increasing Temperature

**Figure 2:**
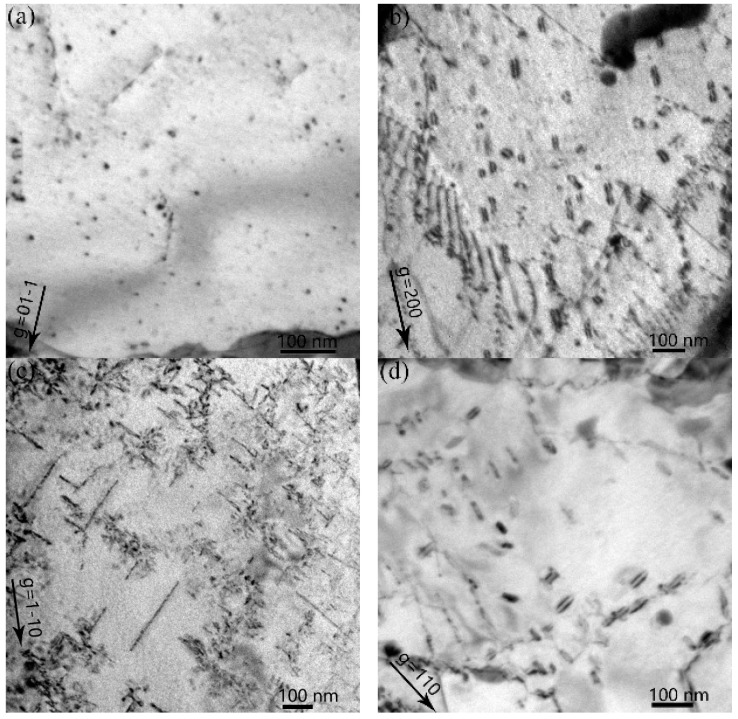
TEM bright field micrographs of specimens irradiated with H^+^ to the fluence of 5 × 10^20^ H^+^ • m^−2^ at elevated temperature ((**a**) 523 K, **g** = 01-1 near the pole [011]; (**b**) 623 K, **g** = 200 near the pole [001]; (**c**) 723 K, **g** = 1–10 near the pole [001]; (**d**) 823 K, **g** = 110 near the pole [001]).

In all of the specimens, no bubbles or cavities were observed by TEM. As shown in Figure 2, interstitial dislocation loops were observed after the hydrogen irradiation to 5 × 10^20^ H^+^ • m^−2^ (0.16 dpa), at temperatures ranging from 523 K to 823 K. In Figure 2a, many small loops, with an average size of 6.6 nm and density of 2.5 × 10^21^ m^−^^3^ (operating diffraction vector (**g**) = 110), were observed after irradiation at 523 K. The Burgers vectors of these dislocation loops were either $\frac{1}{2}\;$a_0_ <111> or a_0_ <100>. The densities of two types of loops were evaluated using the following equations that were derived using the invisibility criteria listed in Table 1:

(1) $$\frac{2}{3}\rho_{b={\;a}_{0}<100>}+\;\frac{1}{2}\rho_{b=\;\frac{1}{2}{\;a}_{0}<111>}={\;\rho }_{g=0-11},$$

(2) $$\frac{1}{3}\rho_{b={\;a}_{0}<100>}+{\;\rho }_{b=\;\frac{1}{2}{\;a}_{0}<111>;}={\;\rho }_{g=-200},$$

ρ**_g_** is the number density of the visible dislocation loops at the different diffraction vector. ρ**_b_** is the number density of the dislocation loops that have the Burgers vector, **b** = $\frac{1}{2}\;$a_0_ <111> or a_0_ <100>. There is an initial approximation that the densities of the loops with all of the possible Burgers vectors with the same family are equal. The density of the loops at the diffraction vectors of **g** = −200 and **g** = 0–11 is $\rho_{g=0-11}$ = 2.5 × 10^21^ m^−3^ and $\rho_{g=-200}$ = 2.76 × 10^21^ m^−3^, respectively. Then, the solution of the equations is $\rho_{b=\frac{1}{2}a_{0}<111>}$ **=** 2.01 × 10^21^ m^−3^ and $\rho_{b=a_{0}<100>}$ **=** 2.24 × 10^21^ m^−3^. The relative proportions of the loops with the Burgers vectors of $\frac{1}{2}\;$a_0_ <111> and a_0_ <100> are 47.3% and 52.7%, respectively.

In Figure 2b, when the temperature increased to 623 K, larger loops with an average size of 34.0 nm and density of 1.1 × 10^21^ m^−3^ (**g** = 110) were observed. When irradiated at 723 K, loops with an average size of 93.6 nm and density of 1.7 × 10^21^ m^−3^ (**g** = 1–10) were observed. Among all of the irradiation temperatures, the loops had the largest average size at 723 K. However, the dislocation loops with a mean size of 36.0 nm and a number density of 7.9 × 10^20^ m^−3^ (**g** = 110) became smaller at a temperature of 823 K. At 623–823 K, all of the loops had the Burgers vectors of **b** = a_0_ <100>.

### 3.2. Dislocation Loop Evolution in RAFM Steel with Increasing Hydrogen Fluence

**Figure 3:**
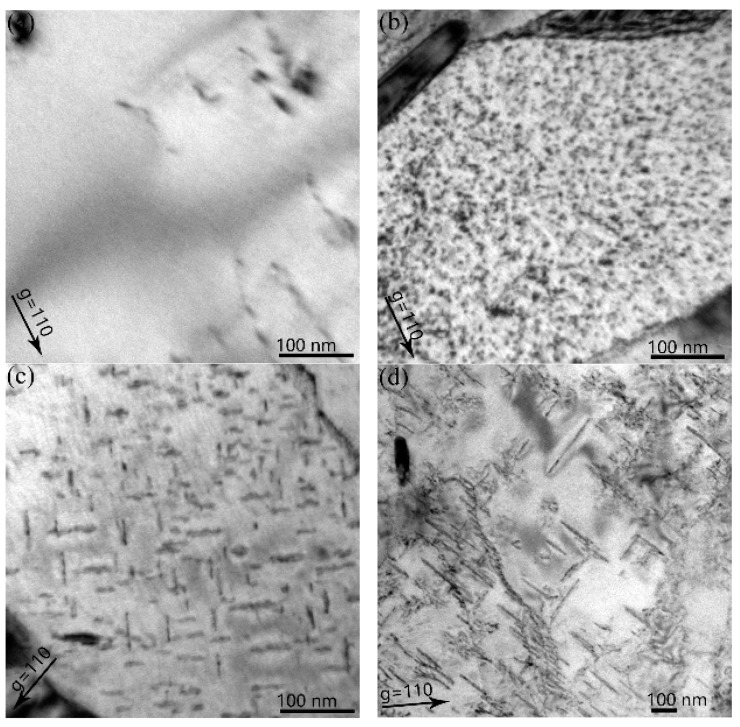
TEM bright field micrographs of specimens irradiated with H^+^ of different fluences at 723 K ((**a**) 1 × 10^20^ H^+^ • m^−2^; (**b**) 2 × 10^20^ H^+^ • m^−2^; (**c**) 3 × 10^20^ H^+^ • m^−2^; (**d**) 5 × 10^20^ H^+^ • m^−2^). All of the micrographs were observed at **g** = 110 near the pole [001].

Figure 3 shows the micrographs of the dislocation loops after hydrogen irradiation at 1 × 10^20^ H^+^ • m^−2^ (0.03 dpa) to 5 × 10^20^ H^+^ • m^−2^ (0.16 dpa), at a temperature of 723 K. At 1 × 10^20^ H^+^ • m^−2^, no dislocation loops were observed in the specimens. However, when the irradiation fluence increased to 2 × 10^20^ H^+^ • m^−2^, small loops with a mean size of 4.3 nm and density of 6.6 × 10^22^ m^−3^ (**g** = 110) were observed. When the irradiation fluence was further increased from 3 × 10^20^ H^+^ • m^−2^ to 5 × 10^20^ H^+^ • m^−2^, the average size and density of the loops increased from 22.9 nm to 93.6 nm and 8.1 × 10^21^ m^−3^ to 1.7 × 10^21^ m^−3^ (**g** = 110), respectively. At 723 K, all of the loops were determined to be **b** = a_0_ <100>.

### 3.3. Burgers Vectors Analysis of Dislocation Loops

**Figure 4:**
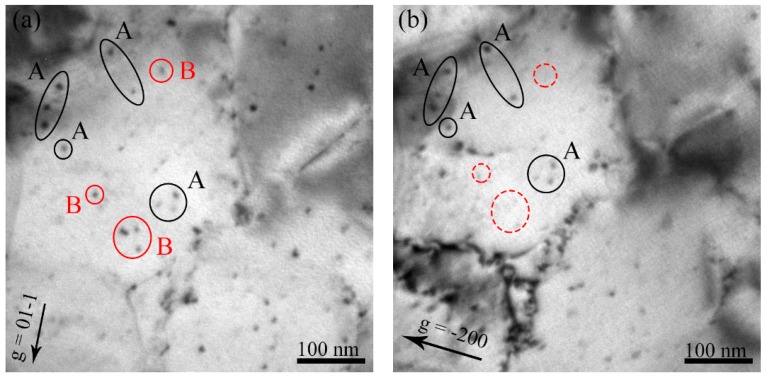
TEM bright field micrographs of dislocation loops in the specimen irradiated to 5 × 10^20^ H^+^ • m^−2^ at 523 K. (**a**) **g** = 01-1, (**b**) **g** = −200 near the pole [011]. The Burgers vector of family A loops is **b** = $\frac{1}{2}\;$a_0_ <111>, which of family B loops is **b** = a_0_ <100>.

**Figure 5:**
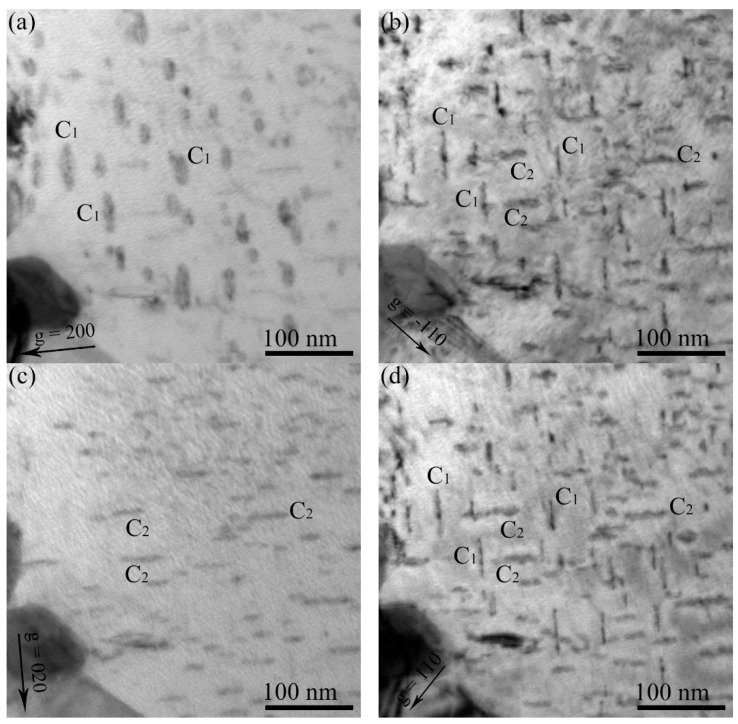
TEM bright field micrographs of dislocation loops in the specimen irradiated at 3 × 10^20^ H^+^ • m^−2^ at 723 K. (**a**) **g** = 200, (**b**) **g** = −110, (**c**) **g** = 020, and (**d**) **g** = 110 near the pole [001]. The Burgers vector of family C_1_ loops is **b** = a_0_ <100>, and the family of C_2_ loops is **b** = a_0_ <010>.

The Burgers vectors of the loops were determined using the invisibility criterion of **g**∙**b** = 0. Figure 4 shows the micrographs of the same area of the specimen irradiated to 5 × 10^20^ H^+^ • m^−2^ at 523 K. The same area of the specimen was captured using **g** = 01-1 and −200 near the pole of [011]. The dislocation loops of family A are present on the **g** of both −200 and 01-1. However, the family B loops are only present on the **g** = 01-1. Therefore, the family A loops were determined to be **b** = $\frac{1}{2}\;$a_0_ <111>, and the family B loops were **b** = a_0_ <100> (Table 1). The dislocation loops in the other specimens were also determined. However, the specimens of the same area were captured using **g** = 200, −110, 020, and 110, close to the pole [001]. Figure 5 shows an example of the Burgers vector analyses of the dislocation loops irradiated at 3 × 10^20^ H^+^ • m^−2^ at a temperature of 723 K. From these four micrographs, it is clear that there are two families of dislocation loops, C_1_ and C_2_. Family C_1_ is only absent in **g** = 020 and C_2_ is only absent in **g** = 200. Therefore, from the Table 1, both the family C_1_ and C_2_ loops were determined to be a_0_ <100>.

## 4. Discussion

### 4.1. Evolution of Dislocation Loops Dependent on Irradiation Temperature

**Figure 6:**
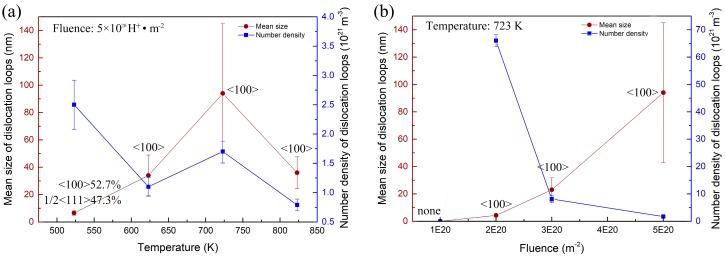
Mean sizes and densities of dislocation loops dependent on irradiation (**a**) temperature and (**b**) fluence. The meaning of the vertical lines is standard deviation.

**Figure 7:**
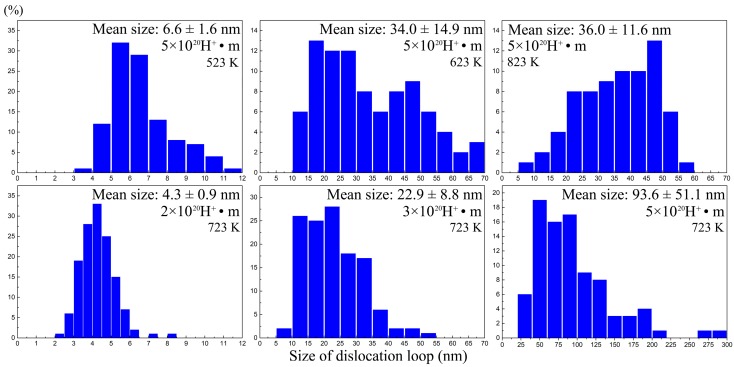
The size distributions of the dislocation loops in all of the specimens. Below each value of the mean size, the corresponding standard deviation is added.

Figure 6 summarizes the evolutions of the loops dependent on the irradiation temperature and fluence, while Figure 7 shows the size distributions of the loops. As shown in Figure 6a, both the size curve and the density curve have a peak at a temperature of 723 K, respectively. This reveals that there is an optimum temperature for dislocation loop growth and nucleation. However, at a low temperature of 523 K, the dislocation loops have the largest number density. This is mostly due to the fact that the diffusion coefficient, D, of the defect (interstitial atom and vacancy) is a function of temperature, as follows:

(3) $$D=D_{0}\;\exp(\frac{-\;Q}{\mathrm{kT}}),$$

where D is the diffusion coefficient of the defect, D_0_ is a pre-factor that is independent of temperature, Q is the activation energy for the migration of the defect, k is the Boltzmann’s constant, and T is temperature. At a low temperature, the diffusion coefficient of the interstitial clusters is small. Thus, the nucleated dislocation loops are hard to migrate, and they combine with each other to grow up. At this stage, the nucleation of the dislocation loops is dominant. With the increase of temperature, the diffusion coefficient of the interstitial clusters increases, so that the dislocation loops can combine with each other to grow up, while the resulting density of the dislocation loop decreases. At 723 K, the nucleation rate and growth rate of the loops reach maximums. But as the temperature is further increased successively, the average size and density of the loops are both decreased. This is ascribed to the fact that vacancy clusters became more mobile. The vacancy has a larger activation energy for migration compared with interstitial atoms. The research of Ehrhart et al. found that the interstitial atoms start to migrate at temperatures below 300 K, but the temperature for the vacancy is above 450 K in pure copper [19]. Therefore, at a high temperature, when the vacancy cluster becomes more mobile, the likelihood of the recombination of vacancy clusters and interstitial clusters will increase. Thus, the average size and number density of the dislocation loops will decrease when the temperature is high enough.

In previous studies, many researchers investigated the relationships between the size and density of dislocation loops and temperature. For example, Klimenkov et al. studied Eurofer-97 irradiated with neutrons at 16.3 dpa, at a temperature of 523 K–723 K. They found that the peak position of the size curve and density curve is at 623 K and 573 K, respectively [20]. In the present study, the correspondent peak temperature is 723 K, which is greater than the results reported in the above literature. On one hand, these differences might be ascribed to the different chemical components of steel, because different types and quantities of solute atoms may change the diffusion coefficient of the defects, and hence change their mobility and recombination. On the other hand, the presence of hydrogen can also significantly change the mobility of the defects, especially for a vacancy. For instance, the migration barrier of a vacancy in α-Fe is 0.64 eV and will be increased to 0.76 eV when the hydrogen is trapped [21]. For F82H, a RAFM steel whose chemical composition is close to the steel that was used in the present study, the migration energy of a vacancy increases from 1.2–1.3 eV to 1.3–1.4 eV after 20 appm of hydrogen is added [22]. This means that upon the presence of hydrogen, a higher temperature is needed for the migration of the vacancy, and hence the recombination of the vacancy with loops. Therefore, the size of the loops shall start to reduce at higher temperatures (i.e., the peak position of the size distribution of dislocation loops with temperature should shift towards higher temperature, just as our experimental results have shown).

### 4.2. Evolution of Dislocation Loop Dependent on Irradiation Fluence

As shown in Figure 6b, with the increasing irradiation fluence of hydrogen, the mean size of the dislocation loops increased, but the number density decreased. This is a universal rule for the dislocation loop evolution dependence of irradiation fluence. What is notable is that the average size of the loops has grown to 93.6 nm and the maximum size is 285.7 nm at a temperature of 723 K. However, the peak damage dose is only 0.16 dpa, but the peak hydrogen atom concentration is as high as 75,500 appm. Previous studies have investigated the evolution of loops in body-centered cubic (bcc) iron and iron–chrome alloys with irradiations of neutron and heavy ion, which is summarized in Table 2 [23,24,25,26,27,28,29,30,31,32,33]. Compared to neutron and heavy ion, we find that hydrogen ion irradiations produce larger dislocation loops at the same damage level. For example, in the literature [28], the average size of the loops is 2.2 nm in F82H with the irradiation of neutrons to 0.7 dpa at a temperature of 523 K. The mean size of the dislocation loop caused by hydrogen irradiation is 6.6 nm (0.16 dpa) at 523 K in the present study, which is much larger than the loops caused by neutron irradiations. This result might be attributed to a high hydrogen concentration in the specimen, because no hydrogen is induced in heavy ion irradiation, and a low hydrogen concentration is induced in the neutron irradiation through the (n, p) transmutation reaction (fission neutron source), while in the present study, a high concentration of hydrogen is implanted into the specimens.

These results indicate that hydrogen has an enhancement effect on the formation of the dislocation loop, especially on the loop growth. The diffusion of hydrogen atoms is definitely fast in the bcc metals. The migration energy of hydrogen in bcc metal is 0.01 eV [34]. However, the hydrogen diffused in the material will be trapped by vacancy clusters and will form stable H–V complexes [35]. The probability of recombination of the self-interstitials with the vacancies that are produced by hydrogen irradiation can be effectively reduced through the formation of H–V complexes [36]. Compared with neutron and heavy ion irradiations, where most of the interstitials and vacancies produced by irradiation will recombine with each other, more interstitials survive under hydrogen irradiation and join in the growth of loops at the same damage level, thus promoting the growth of the dislocation loops.

### 4.3. Burgers Vector Evolution of Dislocation Loop

As shown in Figure 6a, the density ratio of the $\frac{1}{2}\;$a_0_ <111> to a_0_ <100> loops is about 9:10 at 523 K. Upon heating to 623 K and above, only a_0_ <100> loops were observed. The early investigation of the molecular dynamics (MD) simulation found that all of the primary clusters induced by collision cascades in α-Fe are $\frac{1}{2}\;$a_0_ <111> interstitial loops [37,38], and no a_0_ <100> interstitial loops were reported [39]. The work of Masters in 1963 provided the earliest observation of a_0_ <100> loops in iron irradiated with Fe^+^ at 823 K [40]. Therefore, a_0_ <100> loops are considered to stem from $\frac{1}{2}\;$a_0_ <111> loops [14]. However, the mechanisms of the formations of a_0_ <100> loops are still confused. A mechanism, that a_0_ <100> loops come from the transformations of $\frac{1}{2}\;$a_0_ <111> loops through the reaction $\frac{1}{2}\;$a_0_ [111] + $\frac{1}{2}\;$a_0_ [1$\bar{1}\bar{1}$] → a_0_ [100], was reported by Masters from experimental results [26], as well as by Marian et al. and Xu et al. from MD simulations [41,42]. While the research of Chen et al. provided another mechanism, that small a_0_ <100> loops can possibly come from the transformations of $\frac{1}{2}\;$a_0_ <111> loops directly, without the reaction [43]. 

Both of the mechanisms reported above indicate that the mobility of $\frac{1}{2}\;$a_0_ <111> loops may be a critical factor that determines the fraction of $\frac{1}{2}\;$a_0_ <111> transformed to a_0_ <100> loops in the irradiated specimens. Temperature is one of the significant factors affecting the mobility of $\frac{1}{2}\;$a_0_ <111> loops [44]. The mobility of $\frac{1}{2}\;$a_0_ <111> loops is increased at a high temperature, but is immobilized at a low temperature. Therefore, there should be a transition temperature that transforms dominated $\frac{1}{2}\;$a_0_ <111> loops to only a_0_ <100> loops. The transition temperature is about 765 K in pure Fe [24], but is between 523 K and 623 K in Fe-9Cr alloys. The reason for this might be that, besides temperature, solute atom is also a significant factor affecting the transformation ratio of $\frac{1}{2}\;$a_0_ <111> to a_0_ <100> loops. Prokhodtseva et al. investigated pure Fe and Fe(Cr) alloys irradiated with Fe^+^ at 0.05 dpa at room temperature. They found that only the $\frac{1}{2}\;$a_0_ <111> loops were observed in pure Fe, whereas a mixture of $\frac{1}{2}\;$a_0_ <111> and a_0_ <100> loops existed in the Fe(Cr) alloys. This result indicated that Cr favored the formation of a_0_ <100> loops [14]. Hence, the transition temperature should be dropped in the presence of Cr in Fe-based alloys. But the mobility mechanism of the $\frac{1}{2}\;$a_0_ <111> loop seems unfit for the Fe(Cr) alloys [44], because the mobility of the $\frac{1}{2}\;$a_0_ <111> loop decreased owing to the pinning effect of the loop by the added alloying element Cr.

More interestingly, the present study shows that hydrogen favored the formation of a_0_ <100> loops. So far, reports concerning the key parameters (i.e., temperature and dose) for the transformation of $\frac{1}{2}\;$a_0_ <111> to a_0_ <100> loops in RAFM steel are limited. According to the investigation by Wakai et al., a_0_ <100> loops did not appear, and only $\frac{1}{2}\;$a_0_ <111> loops were observed after the F82H was irradiated by neutrons at 2.8 dpa at a temperature of 523 K, and 51 dpa at 573 K [29]. While in the present study, after the RAFM steel specimen was irradiated by hydrogen to the very low dose of 0.16 dpa at 523 K, more than half of the dislocation loops were observed to be the a_0_ <100> type (the density ratio of $\frac{1}{2}\;$a_0_ <111> to a_0_ <100> loops is about 9:10, as shown in Figure 6a). When the hydrogen irradiation temperature increased to 623 K and above at the same dose of 0.16 dpa, only a_0_ <100> loops were observed, while for the neutron irradiation of EUROFER 97 to the dose of 16.3 dpa, no a_0_ <100> loops were observed from 523 to 723 K [20]. Undoubtedly, the present study shows that the presence of hydrogen promoted the formation of a_0_ <100> loops, that is, hydrogen could reduce both the dose and temperature at which a_0_ <100> loops were formed. The mechanism needs further theoretical research from an atomic scale.

## 5. Conclusions

In present study, the evolutions of the dislocation loops that are dependent on irradiation temperature and fluence were investigated using TEM observations in RAFM steel irradiated by hydrogen ions. The main conclusions are summarized as follows:

1. The results of the elevated temperature irradiations indicate that there is an optimum temperature for dislocation loop growth and nucleation, which, in this work, is 723 K. This is because of the differentiation of activation energy for migration between the self-interstitial and vacancy induced by hydrogen and the different chemical components of steels. The presence of hydrogen shifts the peak position of the size distribution of loops with temperature to the higher temperature.
2. Compared with the neutron and heavy ion irradiations at the same damage level, larger dislocation loops were observed in the hydrogen ion irradiation. A potential mechanism was given as follows: the probability of the recombination of self-interstitials with vacancies that are produced by hydrogen irradiation can be effectively reduced through the formation of H–V complexes.
3. At an irradiation temperature of 523 K, 47.3% $\frac{1}{2}\;$a_0_ <111> and 52.7% a_0_ <100> loops were observed, but only a_0_ <100> loops were observed above 623 K, when irradiated to 0.16 dpa. This can be explained by the mechanism where an elevated temperature favors the transformation of $\frac{1}{2}\;$a_0_ <111> to a_0_ <100> loops, and the irradiation ions of hydrogen and solute atoms, such as Cr, might further promote the transformation. The presence of hydrogen promotes the formation of a_0_ <100> loops.

## Figures and Tables

**Table 1:** The invisibility criteria for dislocation loops in TEM observation.

g\b	111	11-1	1-11	−111	100	010	001
0–11	×	√	√	×	×	√	√
−200	√	√	√	√	√	×	×
−110	×	×	√	√	√	√	×
020	√	√	√	√	×	√	×
110	√	√	×	×	√	√	×

Note: **g** is the operating diffraction vector, **b** is the Burgers vector; “√” is visible; “×” is invisible.

**Table 2:** Summary of dislocation loops in pure iron and iron-based alloys under the irradiations of Fe^+^ and neutrons, as well as the present experimental results.

Materials	Irradiation Type	Tirr (K)	Tirr (°C)	Dose (dpa)	Loop Size (nm)		Density (m^−3^)	Burgers Vectors of Loop		Ref.
					Max	Mean		1/2 a_0_ <111>	a_0_ <100>	
Pure Fe	Ion (Fe+)	573	300	1	-	5–20	-	92%	8%	[23]
		673	400	1.3	68	30–50	-	√	√	[24]
		723	450	2	225	-	-	√	√	[24]
		773	500	2	50	-	-	×	√	[24]
				2.5	85	-	-	×	√	[25]
		823	550	74.2	-	100–150	-	×	√	[26]
CLAM	Ion (Fe+)	573	300	0.46	8.5	-	1.9 × 10^22^	-	-	[27]
				2.79	13	-	2.8 × 10^22^	-	-	[27]
		823	550	0.38	18	-	9.4 × 10^21^	-	-	[27]
				2.75	32	-	1.5 × 10^22^	-	-	[27]
F82H	Neutron	523	250	0.7	-	2.2	3 × 10^21^	-	-	[28]
				2.8	-	7.9	1.4 × 10^22^	√	×	[29]
		573	300	51	-	11	4 × 10^22^	√	×	[29]
		575	302	8.8	-	5.4	-	√	√	[30]
		583	310	6.9	-	6.9	2.8 × 10^22^	-	-	[28]
		673	400	7.4	-	33	6 × 10^21^	-	-	[29]
Eurofer-97	Neutron	573	300	15	-	2.8–4.2	2.1–5.8 × 10^21^	√	√	[31]
T91	Neutron	403	130	4.6	-	3.8	2.5 × 10^22^	-	-	[32]
		523	250	8.3	-	4.5	3.6 × 10^22^	-	-	[32]
		633	360	11.8	-	8.9	1.3 × 10^22^	-	-	[32]
FV448	Neutron	653	380	30	110	50	7 × 10^21^	-	>98%	[33]
		733	460	30	>300	300	1 × 10^18^	-	>98%	[33]
Fe-9.24Cr	Ion (H+)	523	250	0.16	11.7	6.6	2.5 × 10^21^	47.3%	52.7%	Present study
		623	350	0.16	68.9	34.0	1.1 × 10^21^	×	√	
		723	450	0.06	8.1	4.3	6.6 × 10^22^	×	√	
				0.1	52.7	22.9	8.1 × 10^21^	×	√	
				0.16	285.7	93.6	1.7 × 10^21^	×	√	
		823	550	0.16	58.1	36.0	7.9 × 10^20^	×	√	

Note: “Tirr” is the irradiation temperature; “-” means that information was given in the reference; “√”, observed; “×”, no observed.
